# HIV-1 integrase resistance associated mutations and the use of dolutegravir in Sub-Saharan Africa: a systematic review and meta-analysis protocol

**DOI:** 10.1186/s13643-020-01356-z

**Published:** 2020-04-25

**Authors:** Ezechiel Ngoufack Jagni Semengue, Maria Mercedes Santoro, Valantine Ngum Ndze, Beatrice Dambaya, Desiré Takou, Georges Teto, Alex Durand Nka, Lavinia Fabeni, Alison Wiyeh, Francesca Ceccherini-Silberstein, Vittorio Colizzi, Carlo-Federico Perno, Joseph Fokam

**Affiliations:** 1 Chantal Biya International Reference Centre for research on HIV/AIDS prevention and management (CIRCB), Yaoundé, Cameroon; 2 Evangelical University of Cameroon, Bandjoun, Cameroon; 3 University of Rome “Tor Vergata”, Rome, Italy; 4 Faculty of Medicine and Biomedical Sciences, University of Yaoundé I, Yaoundé, Cameroon; 5 South African Medical Research Council (MRC), Cochrane South Africa, Pretoria, South Africa; 6 Chair of Biotechnology-UNESCO, University of Rome “Tor Vergata”, Rome, Italy; 7 University of Milan, Milan, Italy

**Keywords:** HIV-1, Integrase, Dolutegravir, Drug resistance, Sub-Saharan Africa

## Abstract

**Background:**

Sub-Saharan Africa carries the greatest burden of HIV-infection with increasing drug resistance burden, which requires improved patient management and monitoring. Current WHO recommendations suggest transitioning to dolutegravir-based (adults) or raltegravir-based-regimens (neonates) for initial antiretroviral therapy (ART) and as a suitable alternative in cases of multi-resistance in resource-limited settings. This review aims at synthesizing the current knowledge on dolutegravir use and integrase resistance-associated mutations found before the wide use of dolutegravir-based regimens.

**Methods:**

This systematic review will include randomized and non-randomized trials, cohort, and cross-sectional studies published on dolutegravir use or integrase resistance-associated mutations in Sub-Saharan Africa. Searches will be conducted (from 2007 onwards) in PubMed, Embase, Cochrane Central Register of Controlled Trials (CENTRAL), Latin American and Caribbean Health Sciences Literature (LILAC), Web of Science, African Journals Online, and Cumulative Index to Nursing and Allied Health Literature (CINAHL) databases. Hand searching of the reference lists of relevant reviews and trials will be conducted and we will also look for conference abstracts. We will include studies of adults and/or children exposed to integrase inhibitors-based therapies; especially dolutegravir or raltegravir (which is our intervention of interest as compared to other antiretroviral regimens). We will exclude studies of patients with specific co-morbidities such as tuberculosis or opportunistic infections. Primary outcomes will be “the rate of viral suppression” and “the level of drug resistance” on integrase inhibitor-based regimens among patients in Sub-Saharan Africa. Secondary outcomes will be “the effect of baseline viremia on viral suppression,” “the effect of treatment duration on viral suppression,” “the proportion of patients with immune recovery,” “the rate of non-adherence,” “rate of adverse events;” “drug resistance according to different integrase inhibitor-based regimens,” and “drug resistance according to viral subtypes/recombinants.” Two reviewers will independently screen titles and abstracts, assess the full texts for eligibility, and extract data. If data permits, random effects models will be used where appropriate. Subgroup and additional analyses will be conducted to explore the potential sources of heterogeneity (e.g., age, sex, baseline viremia, CD4 following treatment, treatment duration, and adherence level).

**Discussion:**

This review will help to strengthen evidence on the effectiveness of integrase strand transfer inhibitors by contributing to current knowledge on the use of dolutegravir and/or raltegravir (especially for neonates) in Sub-Saharan Africa. Results will therefore help in setting-up baseline data for an optimal management of people living with HIV as Sub-Saharan African countries are transitioning to dolutegravir-based regimens. Evidence will also support HIV/AIDS programs in identifying gaps and actions to be undertaken for improved long-term care and treatment of people living with HIV in Sub-Saharan Africa.

**Systematic review registration:**

PROSPERO CRD42019122424

## Background

Integrase strand transfer inhibitors (INSTI) are the latest approved drug class to treat human immune-deficiency virus (HIV) infection. INSTI stop antiretroviral activity by blocking the integration of HIV proviral DNA into the genetic material of host cells. There are currently five drugs in this class; raltegravir (RAL), elvitegravir (EVG), dolutegravir (DTG), cabotegravir (CAB), bictegravir (BIC) [1–7]. These INSTIs are highly effective in both treatment-naïve as well as in treatment-experienced individuals who may harbor multi-drug resistance to other drug classes, with a superior efficacy of DTG, CAB, and BIC both in vitro and in vivo [2, 4, 7]. These latter molecules are known as second generation while RAL and EVG are first-generation INSTI [1, 3, 7–11]. In the group of second-generation INSTI, BIC has been recently approved by the US Food and Drug Administration (FDA) and exist only as part of the fixed dose combination bictegravir/emtricitabine/tenofovir alafenamide (BIC/3TC/TAF) [12, 13]. CAB on its part exists in several formulations and co-formulations and is still in the process for FDA approval [6, 7]. DTG revealed however a slight greater efficacy over other INSTI, due to its low minimal inhibitory concentration, limited risk of cross-resistance with RAL and EVG, better drug tolerance, fewer drug interactions, higher potency, and genetic barrier to resistance than first-generation INSTI [3, 11].

The “Consolidated guidelines on the use of antiretroviral drugs for treating and preventing HIV infection” published in 2016 by the World Health Organization (WHO) clearly recommends early treatment initiation and the use of better-tolerated regimens [11]. However, the ability of HIV to mutate in the course of a failing ART prompts the development of HIV drug resistance and the need of new/more active antiretrovirals such as INSTI. In spite of these benefits, INSTIs and especially DTG are also known to be subject to viral resistance. Of note, more than 40 substitutions have been associated with the development of resistance to INSTIs in HIV-1 B subtypes [2]. The most prevalent mutations are at positions 66, 92, 143, 147, 148, and 155 [2, 14] in the integrase coding region of HIV-1. Of note, G118R is a mutation that has rarely been observed in subtype B viruses, and could have an alternative pathway for DTG-resistance selection in non-subtype B viruses [2]. Furthermore, R263K is preferentially selected among subtype B viruses as compared to other viral subtypes [2]. As viral subtypes (especially B and C) are known to have differential mechanisms in the selection of drug resistance mutations [2], natural polymorphisms in the integrase region may influence the development of resistance against integrase inhibitors in different HIV-1 subtypes [2].

Sub-Saharan Africa (SSA) in particular carries the greatest burden of HIV infection (70% of the global HIV epidemics) with increasing drug resistance burden (about 1/3 of patients). Also, SSA has a very broad HIV genetic diversity (including both types 1 and 2), with an important predominance of HIV-1 non-subtype B viruses that might affect the clinical outcomes in patient management [2]. Several findings support that early treatment with DTG may reduce the emergence of HIV-1 drug resistance (HIVDR) in resource-limited settings (RLS) [15]. These evidences are in line with the WHO recommendations on transitioning to DTG-based regimens for initial option or alternative ART in case of multi-resistance [10, 11]. Up-to-date, few African countries carried out studies on INSTI-resistance. To the best of our knowledge, no systematic review has been done previously to assess the level of resistance to integrase inhibitors across the continent. There is henceforth a need to synthesize the entire knowledge available on integrase resistance-associated mutations found in SSA before the massive adoption of DTG-based regimens. This would help identify the gaps and actions to be undertaken for improved care, follow-up and management of people living with HIV in SSA. The aim of this systematic review is to highlight integrase resistance-associated mutations (i.e., mutations conferring a poor response to an INSTI-based regimen) commonly found in SSA in patients under DTG (or INSTI)-containing regimens. We will first examine the therapeutic outcomes of DTG-use (e.g., viral suppression and immunological response) and the rate of resistance to INSTI among non-suppressive patients. Secondly, we will evaluate (a) the rate of viral suppression according to clinical and biological parameters, (b) the effects of adherence and adverse events on viral suppression, (c) drug resistance and viral susceptibility to each INSTI, and (d) HIV genetic diversity and its effects on acquired INSTI resistance.

## Methods/design

The present protocol has been registered within the PROSPERO database (registration number CRD42019122424 ) and is being reported in accordance with the reporting guidance provided in the Preferred Reporting Items for Systematic Reviews and Meta-Analyses Protocols (PRISMA-P) statement [16] (see checklist in Additional file 1). PRISMA focuses on ways in which authors can ensure the transparent and complete reporting of systematic reviews and meta-analyses.

### Eligibility criteria

#### Design and setting of the study

##### Type of studies to be included

We will include randomized and non-randomized trials, cohort, and cross-sectional studies, evaluating integrase resistance-associated mutations and treatment outcomes in HIV positive patients under DTG containing regimens.

#### Characteristics of the participants

##### Participants

We will include studies of the general population living with HIV, both children and adults who are INSTI-experienced. Studies in patients with HIV-2 will also be included and if HIV type is not specified, we would contact the study authors and request this missing information. The study would be listed as “awaiting classification” while we wait for a response. We will exclude studies of patients with specific co-morbidities such as tuberculosis or opportunistic infections.

##### Intervention

DTG-based regimens will be our intervention of interest. Studies focusing on patients under DTG-based regimens will be considered as our group of interest.

##### Comparators

Given that studies of INSTI-experienced patients will be the only ones included, RAL-based and EVG-based regimens will serve as comparators.

##### Outcomes

Primary outcomes will be “the rate of viral suppression” and “the level of drug resistance” on integrase inhibitor-based regimens among patients in Sub-Saharan Africa. Secondary outcomes will be “the effect of baseline viremia on viral suppression,” “the effect of treatment duration on viral suppression,” “the proportion of patients with immune recovery,” “the rate of non-adherence,” “rate of adverse events;” “drug resistance according to different integrase inhibitor-based regimens,” and “drug resistance according to viral subtypes/recombinants.”

Viral suppression is defined as a plasma viral load less than 1000 copies of viral RNA. The level of resistance refers to the proportion of patients having at least one major INSTI resistance mutation. The immune recovery of a patient refers to the CD4 count > 500 cells/mm^3^ following treatment according to individual studies. Adherence refers to the number of missing dosage as defined by the individual studies. Adverse event refers to side effects of INSTI reported in the individual studies and classified as severe/serious and mere/moderate. Subtypes or recombinants refer to the viral strains identified in a patient sample.

##### Report characteristics

We will restrict the search to articles published in English and French, since the approval of RAL to treat HIV infection in 2007.

#### Information sources and search strategy

We will conduct a comprehensive literature search with the help of two librarians with expertise in systematic reviews.

##### Electronic databases

We will perform the searches in PubMed/MEDLINE, Embase, Cochrane Central Register of Controlled Trials (CENTRAL), Latin American and Caribbean Health Sciences Literature (LILAC) Web of Science, African Journals Online, and Cumulative Index to Nursing and Allied Health Literature (CINAHL).

##### Trial registers

Ongoing trials will be sought in the WHO International Clinical Trials Registry Platform (ICTRP) and ClinicalTrials.gov.

##### Conference abstracts

We will search conference abstract archives on the websites of the Conference on Retroviruses and Opportunistic Infections (CROI); the International AIDS Conference (IAC); the International AIDS Society Conference on HIV Pathogenesis, Treatment, and Prevention (IAS), and all Virology Education conferences, for all available abstracts presented at all conferences from January 2007 onwards.

##### Other sources

Hand searching of the reference lists of relevant reviews and trials will be conducted. In addition, we will contact experts in the field for other potentially eligible studies we may have missed.

The Medical Subject Headings (MeSH terms) for HIV and AIDS and key terms “Integrase”, “Dolutegravir”, “Raltegravir”, “Elvitegravir”, “drug resistance”, “resource-limited settings”, and “Sub-Saharan Africa” will be cross-referenced with terms associated with 56 African countries (Additional file 2 shows the detailed search strategy for Pubmed, Embase, and CINAHL). We will update the search prior to publication to include any additional eligible papers published recently.

### Study records

#### Data management

All records from the various sources included in our search strategy will be combined, uploaded into the reference management software Mendeley (version 1.19.3) and de-duplicated. We will use Microsoft Excel (version 2016 for Windows, Microsoft Corp., Redmond, WA, USA) to record outcomes of the selection process.

#### Selection of eligible studies

Two reviewers (ENJS and BD) will independently screen the titles and abstract to identify potentially eligible studies using an eligibility form. Disagreements will be resolved by consensus, if necessary through discussion with a third reviewer (DT or NAD). The full texts of potentially eligible articles will be obtained and independently reviewed by two authors (ENJS and BD) to identify included studies. Discrepancies will be resolved by consensus and if necessary through discussion with a third reviewer (JF or GT). Studies that are being conducted at the time of the review and which do not yet have results will be identified as ongoing. And studies that have been completed without results could be classified as studies awaiting classification excluded studies and their reasons for exclusion will be described. The PRISMA [16] study flow diagram will reflect this process and detail the reasons for the exclusion of studies.

#### Data collection

We will develop a data extraction sheet to guide data extraction. The sheet will be pilot-tested by two reviewers (VNN and JF) on a random sample of 05 articles and revised as needed. Two reviewers will independently read each eligible full-text article and extract the relevant data. Both sets of data will be entered into Microsoft Excel (version 2016 for Windows, Microsoft Corp., Redmond, WA, USA). Any discrepancies in the extracted data will be resolved by consensus, in discussion with a third reviewer (DT or JF) if necessary.

#### Data items

We will extract the following from included studies:

- Study characteristics (year of publication, study period, study population, study design, aim of study, geographic location, duration of follow-up)
- Study setting (location and type of facilities)
- Characteristics of study population (sample size, age, sex, marital status, weight, enrolment period, inclusion and exclusion criteria)
- INSTI-based regimens
- Duration of INSTI-based regimens
- Viral suppression
- Baseline viral load
- CD4-count following treatment
- INSTI drug resistance
- HIV types and subtypes
- Level of adherence
- Adverse events

There is no pre-planned data assumption.

#### Data synthesis

The main characteristics of all included studies will first be narratively synthesized (i.e., year of publication, study period, study population, study design, aim of study, geographic location, duration of follow-up). A descriptive analysis of study characteristics will be undertaken to explore the heterogeneity of the studies. Summary statistics will then be used to describe study outcomes, including means or medians, and frequencies. Proportions with exact binomial 95% confidence intervals (95% CI) will be calculated for each outcome and presented in forest plots. We will calculate the between-study variance (tau-squared) and *p* values from tests of between-study heterogeneity. We expect substantial between-study heterogeneity, and the focus of the subsequent analyses will therefore be on the identification and exploration of sources of heterogeneity. Finally, we will explore associations that may exist between proportions in countries, settings (e.g., urban, rural), and study outcomes (i.e., the rate of viral suppression and the level of drug resistance) using random intercept logistic meta-regression (binomial-normal) models. These models avoid the biases that arise when normal-normal models are applied to logit or arcsine-square root transformed proportions. Where appropriate, we will use the same models to calculate combined estimates of proportions. We will use the GRADE approach to rate the certainty of evidences as “high,” “moderate,” “low,” and “very low ”[17]. Major findings on INSTI-resistance, drug susceptibility and linkage to DTG-therapy will be summarized in a table. This table will also present the quality of the evidence found, all sorted according to socio-demographic data, clinical, and laboratory parameters. Though the highest quality rating is for randomized trial evidences, they may be downgraded to moderate, low, or even very low-quality evidence. Rating will depend on limitations in study design and implementation, indirectness of evidences, unexplained heterogeneity, imprecision of result, and a high probability of publication bias [17]. Evidences from sound observational studies (cohorts and case-control studies) will be graded as low quality [17]. However, if such studies yield large effects and there is no obvious bias explaining those effects, we would rate the evidences as moderate or—if the effect is large enough—even high quality [17]. Detailed interpretation of each evidence and its respective recommendation is provided in Additional file 3.

### Additional analysis

Subgroup and additional analyses will be conducted following the stratification of the study participants. Results will then be sorted according to age (adults/adolescents vs. children [< 10 years]), sex (male vs. female), baseline viremia, level of CD4 following treatment, INSTI-based regimens, adherence level, and adverse reactions. This will allow us to adjust for potential confounders, to better estimate the effect of each variable on the observed outcomes. If data permits, meta-regression will be performed and summary estimates will help explore the relationship between study-covariates and effect size, in order to highlight any statistical significance.

### Dealing with missing data

If data are missing in key variables, we will contact the study authors for clarifications. A description of missing data will be provided for each study, and we will discuss the possible implications of missing data.

### Assessment of the risk of bias in individual studies

Two reviewers (ENJS and NAD) will assess eligible studies using ROBINS-I [18, 19], a tool for assessing risk of bias in non-randomized studies of interventions. ROBINS provides a systematic way to organize and present the available evidence related to risk of bias in these studies. ROBIS [RoB 2.0] [20, 21] will be used in parallel for randomized studies, which include randomized controlled trials. Risks of bias in cohort and case-control studies will be assessed using the Newcastle-Ottawa scale [22]. These tools will help assess studies but not their outcomes, and will be adapted to the context of this systematic review.

### Meta-biases

Publication bias across individual studies will be assessed by visually inspecting the asymmetry track pattern on the funnel plot; the logistic model will take into consideration study sizes; the quality assessment will be cross-checked, and any disagreements will be resolved within the review team.

### Statistical software

All analyses will be done in Epi info™ version 7 (CDC, USA) and Microsoft Excel version 2016 (Windows, Microsoft Corp., Redmond, WA, USA). Epi info™ will help us calculate means, medians, frequencies, percentages, confidence intervals, and assess primary associations between variables using statistical tests. We will use a validated Excel spreadsheet for meta-analysis and forest plots (https://www.ncbi.nlm.nih.gov/pmc/articles/PMC3296675/), as previously described [23].

## Discussion

This systematic review and meta-analysis will contribute to update the knowledge on integrase resistance-associated mutations found in Sub-Saharan African countries and understand difficulties that may arise with the use of integrase inhibitors before the massive initiation of patients on DTG-based regimens. Our results will firstly be useful for appropriate and contextual management of patients under DTG-based regimens. They would also help to design new strategies in order to improve the care of people living with HIV and reduce the transmission of resistant viruses in the advent of new HIV-infection. This review will thus be highly relevant to inform health system interventions and HIV prevention and treatment strategies in Sub-Saharan Africa, and resource-limited settings in general. As potential limitations of this review, we may be confronted with important study heterogeneity and incompleteness, but these will be considered in statistic models during meta-regression analysis; if not perform, studies incompleteness at least would be solve by contacting study authors. Another limitation may be at the level of reviewing and including studies. In effect, in the process of resolving disagreements while reviewing articles, all team members will be included in the decision-making process or at least aware of the disagreements being discussed. We will try as much as possible to have a consensus decision for each disagreement. Important protocol amendments will be documented, taken into consideration while analyzing the data, and discussed consequently in the final paper. Our findings will be published in a peer-review journal and subsequently disseminated to policy-makers first at the national level through the submission of a governmental notice, and at the international level through conferences and stakeholder meetings.

## Supplementary information

**Additional file 1.** PRISMA-P checklist.

**Additional file 2.** Search Strategy.

**Additional file 3.** Assessing the quality of evidences and the strength of recommendations.

**Additional file 4.** Study eligibility form.

## Data Availability

Data sharing is not applicable to this paper as no datasets were generated or analyzed during the write-up of the current protocol. The latter is however fully available in the PROSPERO repository (https://www.crd.york.ac.uk/PROSPERO/).

## Abbreviations

3TC: Emtricitabine
ART: Antiretroviral therapy
BIC: Bictegravir
CAB: Cabotegravir
CD4: Cluster of differentiation 4
CINAHL: Cumulative Index to Nursing and Allied Health Literature
CROI: Conference on Retroviruses and Opportunistic Infections
DTG: Dolutagravir
EVG: Elvitegravir
FDA: Food and drug administration
HIV/AIDS: Human immunodeficiency virus infection/acquired immune deficiency syndrome
HIVDR: HIV drug resistance
IAC: International AIDS Conference
IAS: International AIDS Society
ICTRP: International Clinical Trials Registry Platform
INSTI: Integrase strand transfer inhibitor
MeSH: Medical Subject Headings
LILAC: Latin American and Caribbean Health Sciences Literature
PRISMA-P: Preferred Reporting Items for Systematic Reviews and Meta-Analyses Protocols
RAL: Raltegravir
SSA: Sub Saharan Africa
TAF: Tenofovir alafenamide
WHO: World Health Organization
