# Supplementary material for: HIV-1 integrase resistance associated mutations and the use of dolutegravir in Sub-Saharan Africa: a systematic review and meta-analysis protocol
Source: Syst Rev. 2020 Apr 25;9:93. doi: 10.1186/s13643-020-01356-z (PMC7183126; doi:10.1186/s13643-020-01356-z)
Supplement: Supplementary file 2 — Additional file 2. Search Strategy. [file 13643_2020_1356_MOESM2_ESM.docx]

**Additional file 2**: Search Strategy

| Database | Query |
| --- | --- |
| PubMed | (((HIV Infections[MeSH] OR HIV[MeSH] OR hiv[tw] OR hiv-1*[tw] OR hiv-2*[tw] OR hiv1[tw] OR hiv2[tw] OR hiv infect*[tw] OR human immunodeficiency virus[tw] OR human immunedeficiency virus[tw] OR human immuno-deficiency virus[tw] OR human immune-deficiency virus[tw] OR ((human immun*) AND (deficiency virus[tw])) OR acquired immunodeficiency syndrome[tw] OR acquired immunedeficiency syndrome[tw] OR acquired immuno-deficiency syndrome[tw] OR acquired immune-deficiency syndrome[tw] OR ((acquired immun*) AND (deficiency syndrome[tw])) OR "sexually transmitted diseases, viral"[MeSH]) OR (Antiretroviral Therapy, Highly Active[MeSH] OR Anti-Retroviral Agents[MeSH] OR Antiviral Agents[MeSH:noexp] OR ((anti) AND (hiv[tw])) OR antiretroviral*[tw] OR ((anti) AND (retroviral*[tw])) OR HAART[tw] OR cART[tw] OR ((anti) AND (acquired immunodeficiency[tw])) OR ((anti) AND (acquired immunedeficiency[tw])) OR ((anti) AND (acquired immuno-deficiency[tw])) OR ((anti) AND (acquired immune-deficiency[tw])) OR ((anti) AND (acquired immun*) AND (deficiency[tw])))) AND ("HIV drug resistance" [Title/Abstract] OR "drug resistance mutations" [Title/Abstract] OR mutation [Title/Abstract] OR resistance [Title/Abstract] OR "resistance associated mutations" [Title/Abstract])) AND ("HIV-Integrase" [Title/Abstract] OR Integrase[Title/Abstract] OR "Integrase strand transfer inhibitors"[Title/Abstract] OR Dolutegravir[Title/Abstract] OR Elvitegravir[Title/Abstract] OR Raltegravir[Title/Abstract] OR "INSTI initiation"[Title/Abstract])) AND ("Africa"[Mesh] OR Africa[Title/Abstract] OR maghreb*[Title/Abstract] OR maghrib*[Title/Abstract] OR Sahara*[Title/Abstract] OR Sub-saharan[Title/Abstract] OR Benin[Title/Abstract] OR Botswana[Title/Abstract] OR Burkina Faso[Title/Abstract] OR Burundi[Title/Abstract] OR "British Indian Ocean Territory"[Title/Abstract] OR Cape Verde[Title/Abstract] OR Canary Islands[Title/Abstract] OR Cameroon[Title/Abstract] OR Ceuta[Title/Abstract] OR Chad[Title/Abstract] OR Comoros[Title/Abstract] OR Eritrea[Title/Abstract] OR Ethiopia[Title/Abstract] OR Equatorial Guinea[Title/Abstract] OR Gabon[Title/Abstract] OR Gambia[Title/Abstract] OR Ghana[Title/Abstract] OR Guinea[Title/Abstract] OR Guinea-Bissau[Title/Abstract] OR Djibouti[Title/Abstract] OR Ivory Coast[Title/Abstract] OR Kenya[Title/Abstract] OR Lesotho[Title/Abstract] OR Liberia[Title/Abstract] OR Libya[Title/Abstract] OR Madeira[Title/Abstract] OR Melilla[Title/Abstract] OR Madagascar[Title/Abstract] OR Malawi[Title/Abstract] OR Mauritius[Title/Abstract] OR Mayotte[Title/Abstract] OR Mozambique[Title/Abstract] OR Mali[Title/Abstract] OR Mauritania[Title/Abstract] OR Namibia[Title/Abstract] OR Niger[Title/Abstract] OR Nigeria[Title/Abstract] OR Reunion[Title/Abstract] OR Rwanda[Title/Abstract] OR Democratic Republic of the Congo[Title/Abstract] OR Seychelles[Title/Abstract] OR South Africa[Title/Abstract] OR Somalia[Title/Abstract] OR Senegal[Title/Abstract] OR Sierra Leone[Title/Abstract] OR Saint Helena[Title/Abstract] OR Sudan[Title/Abstract] OR South Sudan[Title/Abstract] OR Swaziland[Title/Abstract] OR Sao Tome and Principe[Title/Abstract] OR Tanzania[Title/Abstract] OR Togo[Title/Abstract] OR Uganda[Title/Abstract] OR Western Sahara[Title/Abstract] OR Zambia[Title/Abstract] OR Zimbabwe[Title/Abstract])  Filters: Publication date from 2007/01/01 to 2018/12/31 |
| Embase  OvidSP | 1. exp antiretrovirus agent/  2. exp highly active antiretroviral therapy/  3. exp antivirus agent/  4. ((anti and hiv) OR antiretroviral* OR (anti and retroviral*) OR HAART OR (anti and acquired immunodeficiency) OR (anti and acquired immunedeficiency) OR (anti and acquired immuno-deficiency) OR (anti and acquired immune* and deficiency)).tw.  5. or/1-4  6. exp Human immunodeficiency virus infection/  7. (HIV OR HIV 1 OR HIV 2 OR human immunodeficiency virus OR human immuno-deficiency virus OR (human immuno deficiency virus) OR acquired immunodeficiency syndrome OR acquired immuno-deficiency syndrome OR (acquired immuno deficiency syndrome)).tw.  8. or/6-7  9. "HIV drug resistance" OR "drug resistance mutations" OR mutation OR resistance OR "resistance associated mutations".tw.  10. "HIV-Integrase" OR Integrase OR "Integrase strand transfer inhibitors" OR Dolutegravir OR Elvitegravir OR Raltegravir OR "INSTI initiation".tw  11. 5 or 8  12. 9 - 11  13. exp Africa/  14. (Africa OR Maghreb* OR Maghrib* OR Sahara* OR Sub-saharan* OR Benin OR Botswana OR Burkina Faso OR Burundi OR British Indian Ocean Territory OR Cape Verde OR Canary Islands OR Cameroon OR Ceuta OR Chad OR Comoros OR Eritrea OR Ethiopia OR Equatorial Guinea OR Gabon OR Gambia OR Ghana OR Guinea OR Guinea-Bissau OR Djibouti OR Ivory Coast OR Kenya OR Lesotho OR Liberia OR Madeira OR Melilla OR Madagascar OR Malawi OR Mauritius OR Mayotte OR Mozambique OR Mali OR Mauritania2 OR Namibia OR Niger OR Nigeria OR Reunion OR Rwanda OR Democratic Republic of the Congo OR Seychelles OR South Africa OR Somalia OR Senegal OR Sierra Leone OR Saint Helena OR Sudan OR South Sudan OR Swaziland).tw.  15. ("Sao Tome and Principe" OR Tanzania OR Togo OR Tunisia OR Uganda OR Western Sahara OR Zambia OR Zimbabwe).tw.  16. 12 or 13 or 14 or 15  17. 12 and 16  18. limit 17 to yr="2007 - 2018" |
| CINAHL | S1 (MH "Antiretroviral Therapy, Highly Active")  S2 (MH "Anti-Retroviral Agents+")  S3 (MH "Antiviral Agents")  S4 TI ( (anti and HIV) OR antiretroviral* OR (anti and retroviral*) OR HAART OR cART OR (anti and acquired immunodeficiency) OR (anti and acquired immunedeficiency) OR (anti and acquired immuno-deficiency) OR (anti and acquired immun* and deficiency) ) OR AB ( (anti and hiv) OR antiretroviral* OR (anti and retroviral*) OR HAART OR cART OR (anti and acquired immunodeficiency) OR (anti and acquired immunedeficiency) OR (anti and acquired immuno-deficiency) OR (anti and acquired immun* and deficiency)  S5 S1 OR S2 OR S3 OR S4  S6 (MH "HIV Infections+")  S7 (MH "Human Immunodeficiency Virus+")  S8 TI ( HIV OR HIV 1 OR HIV 2 OR human immunodeficiency virus OR human immuno-deficiency virus OR acquired immunodeficiency syndrome OR acquired immunodeficiency syndrome OR (acquired immuno-deficiency syndrome) ) OR AB ( HIV OR HIV 1 OR HIV 2 OR human immunodeficiency virus OR human immuno-deficiency virus OR acquired immunodeficiency syndrome OR acquired immuno-deficiency syndrome or (acquired immuno deficiency syndrome)  S9 (MH "Sexually Transmitted Diseases, Viral+")  S10 S6 OR S7 OR S8 OR S9  S11 TI ("HIV drug resistance" OR "drug resistance mutations" OR mutation OR resistance OR "resistance associated mutations")  S12 TI ("HIV-Integrase" OR Integrase OR "Integrase strand transfer inhibitors" OR Dolutegravir OR Elvitegravir OR Raltegravir OR "INSTI initiation")  S13 S5 OR S10  S14 S11 AND S12 AND S13  S15 (MH "Africa+")  S16 TI (Africa OR Maghreb* OR Maghrib* OR Sahara* OR Sub-saharan* OR Benin OR Botswana OR Burkina Faso OR Burundi OR British Indian Ocean Territory OR Cape Verde OR Canary Islands OR Cameroon OR Ceuta OR Chad OR Comoros OR Eritrea OR Ethiopia OR Equatorial Guinea OR Gabon OR Gambia OR Ghana OR Guinea OR Guinea-Bissau OR Djibouti OR Ivory Coast OR Kenya OR Lesotho OR Liberia OR Madeira OR Melilla OR Madagascar OR Malawi OR Mauritius OR Mayotte OR Mozambique OR Mali OR Mauritania2 OR Namibia OR Niger OR Nigeria OR Reunion OR Rwanda OR Democratic Republic of the Congo OR Seychelles OR South Africa OR Somalia OR Senegal OR Sierra Leone OR Saint Helena OR Sudan OR South Sudan OR Swaziland) OR AB (Africa OR Maghreb* OR Maghrib* OR Sahara* OR Sub-saharan* OR Benin OR Botswana OR Burkina Faso OR Burundi OR British Indian Ocean Territory OR Cape Verde OR Canary Islands OR Cameroon OR Ceuta OR Chad OR Comoros OR Eritrea OR Ethiopia OR Equatorial Guinea OR Gabon OR Gambia OR Ghana OR Guinea OR Guinea-Bissau OR Djibouti OR Ivory Coast OR Kenya OR Lesotho OR Liberia OR Madeira OR Melilla OR Madagascar OR Malawi OR Mauritius OR Mayotte OR Mozambique OR Mali OR Mauritania2 OR Namibia OR Niger OR Nigeria OR Reunion OR Rwanda OR Democratic Republic of the Congo OR Seychelles OR South Africa OR Somalia OR Senegal OR Sierra Leone OR Saint Helena OR Sudan OR South Sudan OR Swaziland)  S17 TI ("Sao Tome and Principe" OR Tanzania OR Togo OR Tunisia OR Uganda OR Western Sahara OR Zambia OR Zimbabwe) OR AB ("Sao Tome and Principe" OR Tanzania OR Togo OR Tunisia OR Uganda OR Western Sahara OR Zambia OR Zimbabwe)  S18 S14 OR S15 OR S16 OR S17  S19 S14 AND S18  S20 S14 AND S18 Limiters - Published Date: 20070101-20181231 |
