# Supplementary material for: HIV-1 integrase resistance associated mutations and the use of dolutegravir in Sub-Saharan Africa: a systematic review and meta-analysis protocol
Source: Syst Rev. 2020 Apr 25;9:93. doi: 10.1186/s13643-020-01356-z (PMC7183126; doi:10.1186/s13643-020-01356-z)
Supplement: Supplementary file 3 — Additional file 3. Assessing the quality of evidences and the strength of recommendations. [file 13643_2020_1356_MOESM3_ESM.docx]

**Additional file 4**: Assessing the quality of evidences and the strength of recommendations.

| **Types of studies** | **Risks of bias** | **Interpretation** | **Quality of evidence** | **Strength of the recommendation** |
| --- | --- | --- | --- | --- |
| Randomized studies | Low risk of bias | Most information is from studies at low risk of bias. | High | Strong |
|  | Unclear risk of bias | Most information is from studies at low or unclear risk of bias. | Moderate | Moderate |
|  |  |  | Low |  |
| Non-randomized studies | High risk of bias | The proportion of information from studies at high risk of bias is sufficient to affect the interpretation of results. | Very Low | Weak |
