# Supplementary material for: HIV-1 integrase resistance associated mutations and the use of dolutegravir in Sub-Saharan Africa: a systematic review and meta-analysis protocol
Source: Syst Rev. 2020 Apr 25;9:93. doi: 10.1186/s13643-020-01356-z (PMC7183126; doi:10.1186/s13643-020-01356-z)
Supplement: Supplementary file 4 — Additional file 4. Study eligibility form. [file 13643_2020_1356_MOESM4_ESM.docx]

**Additional file 4**: Study eligibility form

| **Study/Report ID-**Number: **[Study-ID number:** Use the code from the **EXCEL-Sheet**] | |
| --- | --- |
| **STUDY PERIOD / PUBLICATION DATE** 1. Study period after **2007** (RAL introduction) | YES UNCLEAR NO  **EXCLUDE** |
| **PARTICIPANTS / TARGET** 2. HIV-1-infected patients | YES UNCLEAR NO  **EXCLUDE** |
| **SETTING** 3. People living with HIV in sub-Saharan AFRICA | YES UNCLEAR NO  **EXCLUDE** |
| 4. “specific population” (Is a specific sub population studied such as PMTCT, children/adolescents, child-bearing age, pregnant women, Men, drug resistance) | YES UNCLEAR NO  **EXCLUDE** |
| **OUTCOMES** 5. At least ONE element of the following is measured: a. level of resistance ☐ b. effectiveness of the drug ☐ c. ART policies ☐ d. On INSTI ☐ e. Virally suppressed ☐ | YES UNCLEAR NO  **EXCLUDE** |
| **STUDY DESIGNS or PAPER TYPE** 6. At least ONE of the following is concerned: a. Cohort study ☐ b. Cross sectional study ☐  c. Clinical trial ☐  d. Report / Clinical guideline ☐  e. Governmental paper ☐  f. Academic Thesis ☐  g. Systematic review ☐ | YES UNCLEAR NO  **EXCLUDE** |
| **FINAL DECISION** 1x EXCLUDE = EXCLUDE 1x UNCLEAR = UNCLEAR | YES UNCLEAR NO  **EXCLUDE** |
| **NOTES: Unclear papers are to be assessed like disagreements!** | |
